# Unaffected Li-Fraumeni Syndrome Carrier Parent Demonstrates Allele-Specific mRNA Stabilization of Wild-Type *TP53* Compared to Affected Offspring

**DOI:** 10.3390/genes13122302

**Published:** 2022-12-07

**Authors:** Jeffrey S. Buzby, Shirley A. Williams, Diane J. Nugent

**Affiliations:** 1 Hematology Research and Advanced Diagnostics Laboratories, CHOC Children’s Hospital of Orange County, Orange, CA 92868, USA; 2 Division of Hematology, CHOC Children’s Hospital of Orange County, Orange, CA 92868, USA; 3 Division of Pediatric Hematology, School of Medicine, University of California at Irvine, Orange, CA 92868, USA

**Keywords:** p53, Li-Fraumeni Syndrome, tumor suppression, cancer inheritance, allele-specific expression, mRNA stability

## Abstract

Li-Fraumeni Syndrome (LFS) is an autosomal dominant disorder where an oncogenic *TP53* germline mutation is inherited by offspring of a carrier parent. p53 is a key tumor suppressor regulating cell cycle arrest in response to DNA damage. Unexpectedly, some mutant *TP53* carriers remain unaffected, while their children develop cancer early in life. To begin unravelling this paradox, the response of dermal fibroblasts (dFb) isolated from a child with LFS was compared to those from her unaffected father after UV exposure. Phospho-Chk1[S345], a key activator of cell cycle arrest, was increased by UV induction in the LFS patient compared to their unaffected parent dFb. This result, along with previous findings of reduced *CDKN1A*/p21 UV induction in affected dFb, suggest that cell cycle dysregulation may contribute to cancer onset in the affected LFS subject but not the unaffected parent. Mutant p53 protein and its promoter binding affinity were also higher in dFb from the LFS patient compared to their unaffected parent. These results were as predicted based on decreased mutant *TP53* allele-specific mRNA expression previously found in unaffected dFb. Investigation of the potential mechanism regulating this *TP53* allele-specific expression found that, while epigenetic promoter methylation was not detectable, *TP53* wild-type mRNA was specifically stabilized in the unaffected dFb. Hence, the allele-specific stabilization of wild-type *TP53* mRNA may allow an unaffected parent to counteract genotoxic stress by means more characteristic of homozygous wild-type *TP53* individuals than their affected offspring, providing protection from the oncogenesis associated with LFS.

## 1. Introduction

The tumor suppressor p53 is a key transcriptional regulator of the cellular response to environmental, genotoxic stress [1]. Consequently, over 50% of all human cancer cases can be attributed to an acquired loss-of-function (LOF), with missense *TP53* mutations resulting in impairments to adequately repair DNA damage, regulate cell division, and/or effectively eliminate cancer cells through apoptosis [2]. While spontaneous mutations have been detected throughout the *TP53* gene, the majority of cancerous mutations are localized to its DNA-binding domain, encoded by exons 5–8 [3].

Li-Fraumeni Syndrome (LFS) is a rare, autosomal dominant disorder where a *TP53* germ line mutation can be inherited by the offspring of a carrier parent [4]. Carriers of such mutations have a predisposition for the early onset of cancer, with 68% of males and 93% of females developing some form of cancer in their lifetimes. Additionally, the average age of cancer onset is significantly earlier in LFS patients, with 56% of cases occurring before age 30 and 100% before age 50, compared to only 2% and 11%, respectively, in the general population [4].

Although many studies have characterized tumors with acquired p53 mutations, data on the prevalence of cancer in LFS family members carrying the same germ line mutant *TP53* allele but remaining unaffected by cancer is much more limited [5,6]. Likewise, the regulatory factors contributing to the absence of cancer in these carriers also remain largely unknown. Yet, the reason why some of these *TP53* mutation carriers do not develop cancer may profoundly impact the future directions of cancer detection, targeted therapy, and patient survival [7,8]. Our goal is to identify these differences between affected and unaffected LFS family members and characterize the mechanisms that either protect carriers or promote oncogenesis in their affected offspring.

Thus, we hypothesized that studies of LFS families with both affected and unaffected members may illuminate their mutation-driven responses to reveal possible points of intervention for preventing oncogenesis. Family studies such as this benefit from the family members’ otherwise similar genetic backgrounds to facilitate focus on the cancer-related variations. To this end, we have identified a highly informative LFS family in which the father remains cancer-free in his fourth decade of life [9]. He only became aware of his *TP53* mutation when he was screened after his two children developed choroid plexus carcinomas before the age of 4. At that time, he was found to carry a de novo Ser241Tyr C > A mutation in exon 7 of *TP53* that was inherited by his offspring, who eventually succumbed to cancer. Their mother has no *TP53* nor any other relevant, pathogenic mutations.

Previous efforts to examine the impact of p53 mutations on its downstream regulatory pathways [10] have been very useful for elucidating their effects on cell cycle arrest [11] and DNA repair [12]. Many of these studies have also focused on investigating these cellular responses to UV irradiation mediated by p53 and its mutants in vitro [13,14,15,16,17]. Hence, primary dermal fibroblast (dFb) cultures were established using tissue samples obtained from the affected LFS daughter and her unaffected father to begin comparing their in vitro responses to UV stress in our previous study [18]. It was hypothesized that the unaffected carrier father may counteract genotoxic stress by means more characteristic of homozygous wild-type *TP53* individuals than that of his affected offspring. In support of this hypothesis, the previous report of our initial studies [18] presented evidence that UV induction of *CDKN1A*/p21 was significantly increased [17], while the phosphorylation of p53[Ser15] was significantly decreased [19] in dFb from the LFS unaffected carrier compared to his affected offspring. Both these responses of the unaffected dFb were, in fact, more comparable to those of homozygous wild-type *TP53* control dFb. Based on these results, it was further hypothesized that the unaffected dFb may not express as much of the mutant *TP53* allele as the affected dFb. An analysis of their *TP53* allele-specific expression found that this was indeed the case [18].

The studies presented here are designed to further characterize regulation of both UV-induced cell cycle arrest [17] and *TP53* allele-specific expression [20] in dFb from the unaffected LFS carrier compared to his affected offspring [18]. Comparing the genotoxic response profiles of these heterozygous *TP53* family members offers a unique opportunity to gain insight into the potential protective vs. oncogenic components involved in p53-mediated tumorigenesis. A greater understanding of this tumor suppression mechanism may uncover unique targets for developing new therapeutic agents to prevent cancer [7,8].

## 2. Materials and Methods

### 2.1. Culture, Irradiation, and Harvest of Human Dermal Fibroblasts

Proliferating cultures of human dFb were established from fresh LFS family tissue samples [18], as described [21], with expression of Fibroblast-Specific Protein-1 (FSP-1)/S100A4 [22] confirmed. Control dFb were derived from unrelated foreskin samples [23]. Cultures were grown, maintained, and experimentally utilized as previously described [18].

Using previously optimized UV irradiation conditions [18], dFb six-well plate cultures were aspirated of media, washed with PBS, then immediately challenged by exposure to UVC (254 nm) radiation (1 mJ/cm^2^ = 10 J/m^2^) in a GS UV Chamber (Bio-Rad, Hercules, CA, USA), directly followed by media replenishment.

Total RNA was isolated using RNeasy Mini Kits (QIAGEN, Germantown MD USA) and quantified using RNA 6000 Nano Kits with a BioAnalyzer 2100 (Agilent, Santa Clara, CA, USA).

Genomic DNA (gDNA) was isolated using Blood & Cell Culture DNA Midi Kits (QIAGEN) and quantified with a NanoDrop ND-1000 Spectrophotometer (ThermoFisher Scientific, Waltham, MA, USA).

Cellular protein was extracted in PathScan^®^ Lysis Buffer (Cell Signaling Technology, Danvers, MA, USA); supplemented with additional phosphatase and protease inhibitors (20 mM Tris-HCl pH 7.5, 150 mM NaCl, 1% Triton, 25 mM sodium fluoride, 1 mM β-glycerophosphate, 1 mM EGTA, 1.5 mM EDTA, 30 mM sodium pyrophosphate, 4 mM Na_3_VO_4_; 1 µM okadaic acid, 1X-Protease Inhibitor Cocktail (Cell Signaling Technology), 1 mM PMSF, and 20 U/mL DNase I). Protein content was determined using the modified Lowry, DC Protein Assay (Bio-Rad, Hercules, CA, USA).

### 2.2. Immunoblot Analysis 

Protein extracts (30 µg/lane) were electrophoresed and electroblotted as previously described [18]. Electroblotted membranes were blocked with 5% non-fat milk (Nestlé Carnation, Solon, OH, USA) in TBS (Cell Signaling Technology, Boston, MA, USA) for 60 min at room temperature. They were then incubated with either primary 1:1000 antiphospho-Chk1[S345] (Cell Signaling Technology, #2348), 1:1000 anti-Chk1 (Cell Signaling Technology; #2360), 1:1000 anti-phospho-Chk2[T68] (Cell Signaling Technology; #2197), 1:1000 anti-Chk2 (Cell Signaling Technology; #6334), 1:500 anti-mutant p53 (abcam, Boston, MA, USA; #ab32049), 1:1000 anti-p53 (Santa Cruz Biotechnology, Dallas, TX, USA; #sc-6243), or 1:5000 anti-β-actin (Santa Cruz Biotechnology; #sc-47778) overnight at 4°C, followed by the corresponding horseradish peroxidase-conjugated goat anti-rabbit (Cell Signaling Technology; #7074) or anti-mouse (Cell Signaling Technology; #7076) secondary IgG for 2 h at room temperature in TBST (Cell Signaling Technology) with 5% non-fat milk (Nestlé Carnation) or 5% BSA (Cell Signaling Technology), according to the antibody manufacturer’s recommendations. Washed membranes were developed for optical density quantitation of chemifluorescent signals from replicate immunoblots as previously described [18,24].

### 2.3. p53 Promoter Binding Activity

The TF-Detect Human p53 Activity Assay Kit (GeneCopoeia, Rockville, MD, USA) was used to evaluate p53 interaction with a consensus promoter binding site. After UV irradiation of confluent dFb cultures, nuclear protein extracts were prepared according to the kit manufacturer’s recommendations. Equal amounts of nuclear protein (2 µg/well) from the affected LFS, unaffected, or control dFb were then assayed according to the kit manufacturer’s specifications, along with a provided recombinant p53 standard to compare the amount of binding activity in each sample.

### 2.4. Cytosine Methylation Analysis of TP53 Gene Promoter

gDNA isolated from proliferating cultures of control, unaffected, and affected dFb was subjected to chemical conversion of unmethylated cytosines into uracils by bisulfite treatment using the EpiMark Bisulfite Conversion Kit (New England BioLabs, Ipswich, MA, USA). The *TP53* gene promoter region from −1654 to +54 was then amplified as six overlapping PCR products of 328, 338, 396, 385, 423, and 326 base pairs, respectively, using custom primers specific for bisulfite-converted gDNA (IDT, San Diego, CA, USA). These six overlapping fragments of the *TP53* gene promoters were subjected to Sanger DNA sequencing analysis to identify unconverted 5-methyl-/hydroxymethyl-cytosines (Retrogen, San Diego, CA, USA).

### 2.5. Reverse-Transcribed (RT)-qPCR mRNA Expression

RT-qPCR (two-step) utilized iScript Advanced cDNA Synthesis Kits (Bio-Rad) with 25 ng RNA/sample, followed by the SsoAdvanced Universal SYBR Green Supermix (Bio-Rad) on a CFX Connect Real-Time PCR System (Bio-Rad). Validated qPCR primers specific for human gene transcripts of *TP53* (p53, Bio-Rad; qHsaCID0013658), *S100A4* (FSP-1, Bio-Rad; qHsaCID0013749), and the reference standard, *HPRT1* (hypoxanthine phosphoribosyltransferase 1, Bio-Rad; qHsaCID0016375), were utilized according to the manufacturer’s specifications (Bio-Rad). Reference gene-normalized relative gene expression was calculated based on ΔΔC_q_ values by the CFX Manager 3.1 operational software (Bio-Rad).

### 2.6. TP53 mRNA Half-Life

Confluent cultures of control, unaffected, and affected dFb were exposed to 10 µg/mL of the transcriptional inhibitor, actinomycin D (Life Technologies, Carlsbad, CA, USA) 24 h after UV irradiation, as previously described [25]. Cultures were then harvested and their cytoplasmic RNA isolated at 0, 120, 180, 240, and 360 min after inhibition of transcription. The RNA was subjected to RT-qPCR analysis for *TP53* mRNA stability over time, with *HPRT1* mRNA as a stable reference standard. After normalizing to the *HPRT1* mRNA standard, the fraction of *TP53* mRNA remaining at each time point after inhibition of transcription was calculated relative to that of the respective samples prior to inhibition (time = 0 h). These values were then plotted against the time after inhibition of transcription with Prism V.3 (GraphPad Software, San Diego, CA, USA) to determine *TP53* mRNA half-life based on its exponential decay, as described [26].

### 2.7. RT-PCR-RFLP Analysis for TP53 Allele-Specific mRNA Stability 

RNA isolated from UV-irradiated control, unaffected, and affected dFb cultures, 0 and 360 min after inhibition of transcription in the experiment described above, was further evaluated for *TP53* allele-specific mRNA stability by slight modifications of our previously developed procedure [18,27]. To monitor transcript decay, a 114 bp region of *B2M* (β-2-microglobulin) was also amplified using custom, intron-spanning primers; 5′-ACCCCCACTGAAAAAGATGA-3′ and 5′-ATCTTCAAACCTCCATGATG-3′ (QIAGEN), as a stable reference standard [23,28]. PCR amplification, *Sbf*I (New England BioLabs, Ipswich MA USA) digestion, and RFLP product quantitation were all carried out as previously described [18]. After PCR amplification, expression of each reverse-transcribed *TP53* allele transcript was calculated by normalizing molar ratios of the 75 bp *Sbf*I RFLP product from the wild-type *TP53* allele and the uncleaved 94 bp product from the mutant allele to that of the 114 bp *B2M* reference amplicon for each sample. The fraction of each allele remaining 6 h after inhibition of transcription was then calculated relative to that of their respective samples prior to inhibition (time = 0 h).

### 2.8. Statistics

Data was presented as mean ± standard-error-of-the-mean (SEM) from four or more sample replicates. Probability of significant differences (*p* < 0.05) between two data sets was calculated using Student’s *t*-test on InStat V.3 for Windows (GraphPad Software).

## 3. Results

### 3.1. Phospho-Chk1[S345] Induction Was Significantly Upregulated by UV Exposure in Affected LFS Fibroblasts

Two complementary systems that respond to DNA damage in human cells are triggered by the protein kinase sensors, ATR and ATM [29]. Key cell cycle checkpoint kinases 1 (Chk1) and 2 (Chk2) are then directly phosphorylated as signal transduction substrates of ATR and ATM, respectively [30]. p53 acts further downstream in both the ATR- and ATM-activated pathways, and both these kinase sensors can also themselves phosphorylate p53[S15] upon activation [31]. With regards to our previous data showing significantly greater UV-induced phospho-p53[S15] in the affected LFS dFb [18], the ATR kinase-activated pathway is widely considered to be more responsive to UV-induced damage [31,32], though there can be some crosstalk between the two sensors in their damage detection [29]. Interestingly, it has been shown that phospho-p53[S15] may play a role in sustaining activated phospho-Chk1[S345] as well [33].

**Figure 1:**
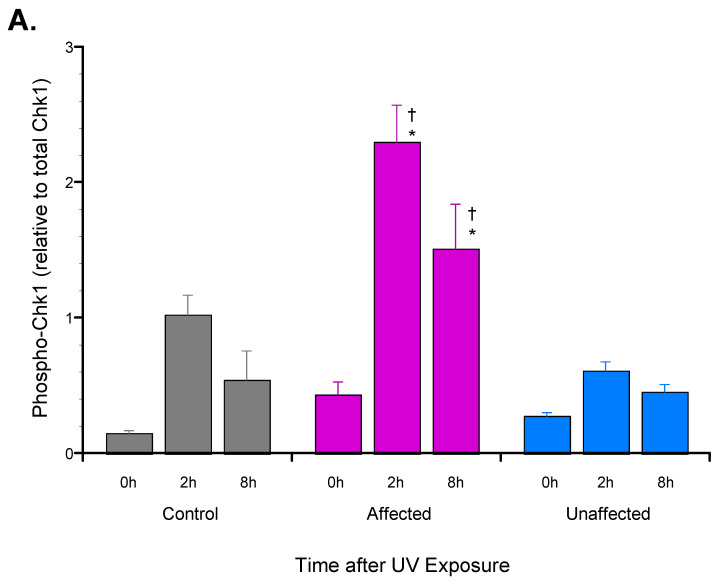

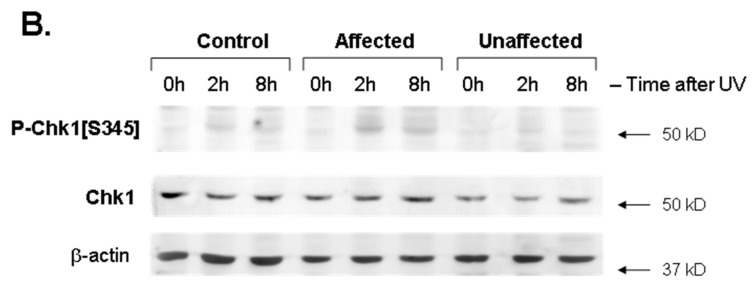
Phospho-Chk1[S345] induction by dFb from affected LFS compared to unaffected or control subjects in response to UV exposure. Protein extracts of cultured dFb were subjected to immunoblot analysis following UV irradiation, as described. (**A**) Chemifluorescent signals corresponding to phospho-Chk1[S345] (56 kD) were normalized to those of total Chk1 protein (56 kD) following UV irradiation for each dFb source (*n* = 6: * *p* < 0.05, affected vs. unaffected dFb; ^†^ *p* < 0.05, affected vs. control dFb). (B) Results from representative immunoblot.

Based on these previous observations, UV-induced S345-phosphorylation of Chk1 was compared in dFb from the affected LFS subject vs. both the unaffected parent and the unrelated control subject using immunoblot analysis. Phospho-Chk1[S345] was significantly increased in dFb protein extracts from the affected LFS subject compared to both the unaffected parent and the unrelated control subject, 2–8 h after UV exposure (Figure 1). Parallel immunoblot analysis of Chk2[T68] phosphorylation found no significant UV-induced response in any of the dFb tested. As was somewhat expected [29,31,32], these results indicated that the UV irradiation conditions under study primarily activated the ATR-Chk1, as opposed to the ATM-Chk2, pathway [30]. Furthermore, they also suggested that our previously observed significant increase in UV-induced phospho-p53[S15] by the affected dFb [18] may contribute to this concomitantly increased UV-induced phospho-Chk1[S345], based on their reported cooperativity [33].

### 3.2. Mutant p53 Protein Expression Was Significantly Reduced in Unaffected LFS Fibroblasts

To determine if the decreased mutant *TP53* allele-specific mRNA expression reported previously in the unaffected dFb [18] translated into decreased expression of mutant p53 protein as well, mutant p53 UV induction was compared in dFb from the affected LFS subject vs. the unaffected parent and the unrelated control subject using immunoblot analysis with an antibody (anti-mp53) developed to detect mutant p53 (mp53). While not exclusively specific to the Ser241Tyr mutation in the present study, this anti-mp53 was characterized by the manufacturer to detect six other p53 mutants within an 80 amino acid region of the DNA-binding domain surrounding Ser241Tyr [3] and not to detect wild-type p53.

**Figure 2:**
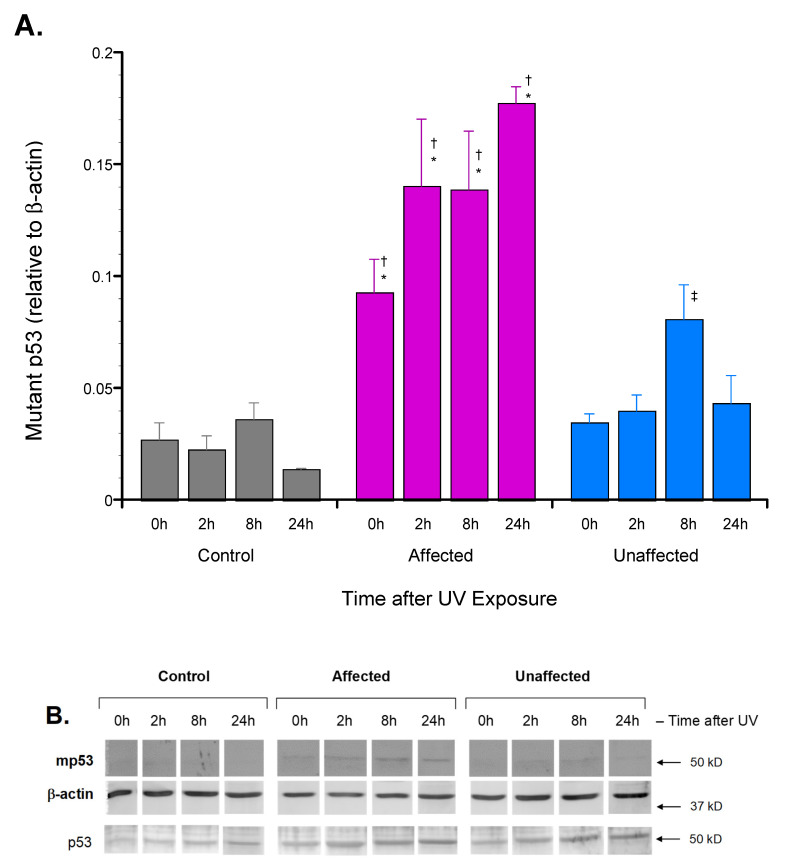
Mutant p53 (mp53) protein induction by dFb from affected LFS compared to unaffected or control subjects in response to UV exposure. Protein extracts of cultured dFb were subjected to immunoblot analysis following UV irradiation, as described. (**A**) Chemifluorescent signals corresponding to mp53 (53 kD) were normalized to those of β-actin (43 kD) following UV irradiation for each dFb source (*n* = 8: * *p* < 0.05 affected vs. unaffected dFb; ^†^ *p* < 0.05, affected vs. control dFb; ^‡^ *p* < 0.05 unaffected vs. control dFb). (**B**) Results from representative immunoblot.

Immunoblot analysis found mp53 expression by the affected dFb to be significantly greater than that of both the unaffected and wild-type control dFb at all time points 0–24 h after UV irradiation (Figure 2). While mp53 expression was also detectable in the unaffected dFb above the basal level of the unrelated wild-type control dFb (Figure 2B), it was only significantly higher than the wild-type control dFb at the 8 h time point after UV irradiation (Figure 2A). Hence, these results were as predicted based on the decreased mutant *TP53* allele-specific mRNA expression previously reported for the unaffected dFb [18].

### 3.3. p53 Promoter Binding Activity Was Significantly Upregulated by UV Exposure in Affected LFS Fibroblasts

**Figure 3:**
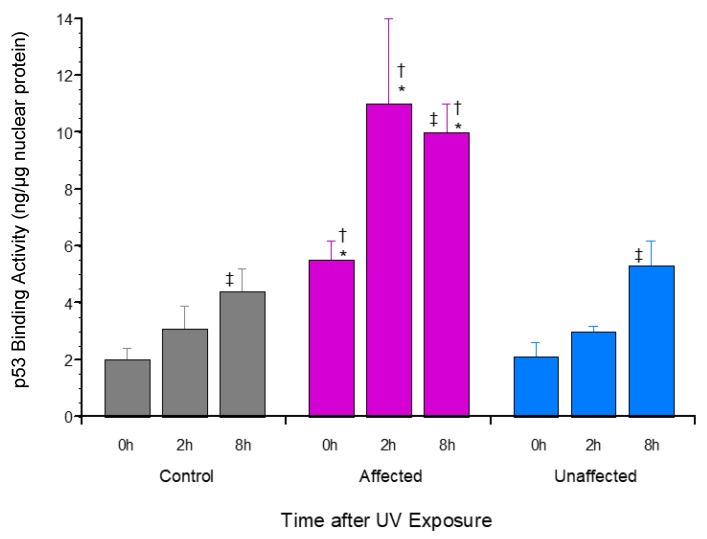
p53 promoter binding activity by dFb from affected LFS compared to unaffected or control subjects in response to UV exposure. TF-Detect Human p53 Activity Assay Kit (GeneCopoeia, Rockville, MD, USA) was used to compare amount of consensus p53 promoter binding site activity in nuclear protein extracts from proliferating dFb ±UV irradiation, according to manufacturer’s recommendations (*n* = 4: * *p* < 0.05, affected vs. unaffected dFb; ^†^ *p* < 0.05, affected vs. control dFb; ^‡^ *p* < 0.05, vs. -UV irradiation (time = 0 h)).

In addition to this significantly increased mp53 expression (Figure 2), significantly greater UV-induced p53[S15] phosphorylation was also reported [18] in affected dFb protein extracts, as discussed above relative to phospho-Chk1[S345]. Since both p53 phosphorylation [19,34] and mutation [35,36] have been found to potentially modify interaction with its various effector promoter binding sites, the affinity of UV-induced dFb nuclear extracts from the affected LFS vs. both the unaffected parent and unrelated control subjects was compared using an activity assay to measure p53 bound to a consensus promoter binding site [37]. The results of this assay found that affected dFb protein extracts had significantly higher p53 binding with the target consensus promoter element than that of the unaffected parent and the unrelated control subject dFb both before and 2–8 h after UV exposure (Figure 3). This finding suggested that the increased mp53 (Figure 2) in the affected dFb may alter its evident promoter binding affinity and/or specificity compared to wild-type p53 (Figure 3).

### 3.4. Cytosine Methylation of TP53 Gene Promoters Was Not Detected

It was hypothesized that genomic imprinting [38] via differential *TP53* promoter methylation [39] may regulate its previously reported allele-specific expression in dFb from the unaffected parent [18]. To address this possibility, gDNA isolated from proliferating cultures of control, unaffected, and affected dFb was subjected to bisulfite conversion of unmethylated cytosines to uracils. Their *TP53* gene promoter regions from −1654 to +54 were then DNA sequenced to identify any unconverted 5-methyl-/hydroxymethyl-cytosines.

Methylation of this promoter region was not detected for gDNA from any of these subjects, despite the presence of a canonical CpG island [39]. While there are reports of *TP53* promoter methylation, its consistent role in *TP53* regulation has yet to be established [39]. Indeed, there are other studies that did not detect its methylation either [40,41], one of which was also focused on LFS familial inheritance [41].

### 3.5. TP53 mRNA Was Significantly More Stable in Unaffected LFS Fibroblasts

**Figure 4:**
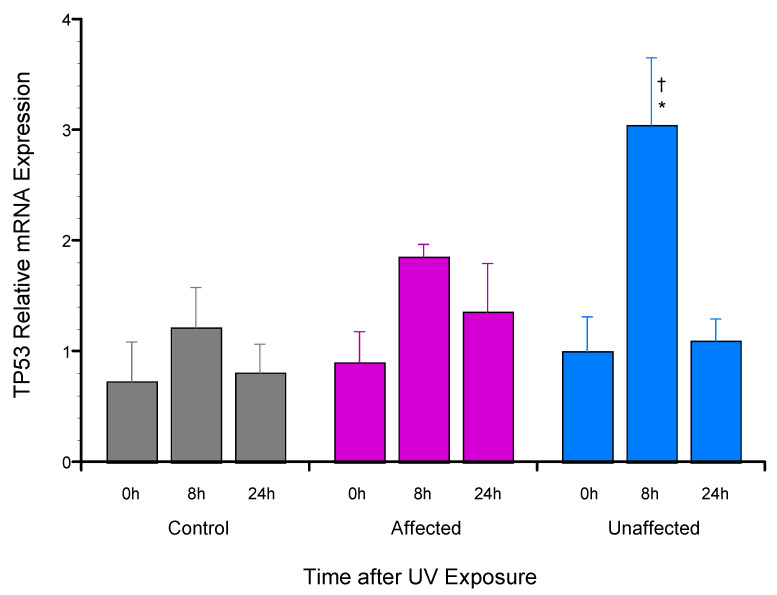
*TP53* mRNA expression by dFb from unaffected compared to affected LFS or control subjects in response to UV exposure. Total dFb RNA was analyzed by RT-qPCR following UV irradiation, as described. Expression of *TP53* mRNA normalized relative to *HPRT1* mRNA was calculated based on ΔΔC_q_ values at each sample time point (*n* = 4: * *p* < 0.05, affected vs. unaffected dFb; ^†^ *p* < 0.05, affected vs. control dFb).

To investigate potential molecular mechanisms regulating the previously reported *TP53* allele-specific expression in the unaffected dFb [18], the expression of total *TP53* mRNA was first compared by RT-qPCR analysis following UV exposure. This comparison found significantly increased total *TP53* mRNA expression in the unaffected dFb vs. both affected and control dFb 8 h after UV exposure (Figure 4). 

**Figure 5:**
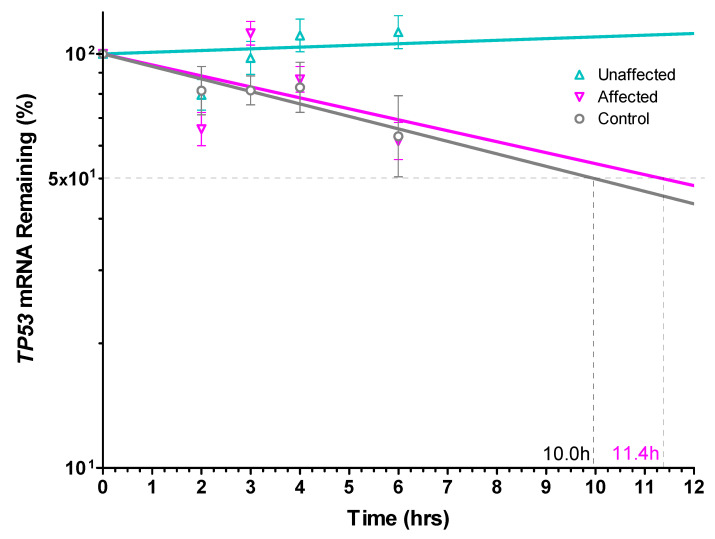
*TP53* mRNA half-life by dFb from unaffected compared to the affected LFS or control subjects following UV exposure. Total dFb RNA was analyzed by RT-qPCR following transcriptional inhibition with actinomycin D for the time periods indicated after UV irradiation, as described. Expression of *TP53* mRNA normalized relative to *HPRT1* mRNA as a stable reference standard was calculated based on ΔΔC_q_ values at each sample time point (*n* = 5). mRNA half-life (time after which 50% of initial *TP53* mRNA remains) was calculated by curve fit regression analysis with Prism V.3 (GraphPad Software). (**∆**) Unaffected, (**∇**) Affected, and (**○**) Control.

A prominent role for mRNA stability in regulating *TP53* expression has been described [42,43,44], often superseding transcriptional control [45,46]. Consequently, the total *TP53* mRNA half-life was compared in UV-induced dFb from the unaffected parent vs. the affected LFS and unrelated control subjects to determine its possible contribution to the higher UV-induced total *TP53* mRNA expression found in the unaffected dFb (Figure 4). The results of this study found a comparable 10–11 h transcript half-life in both affected and control dFb (Figure 5). This relatively long *TP53* mRNA half-life is consistent with that reported previously in other cells [42,43,46]. However, the UV-induced *TP53* mRNA expressed by the unaffected dFb was found to be completely stable throughout the duration of this study (Figure 5). This higher *TP53* mRNA stability very likely plays a role in the observed upregulation of the total *TP53* mRNA expression in the unaffected dFb (Figure 4).

**Figure 6:**
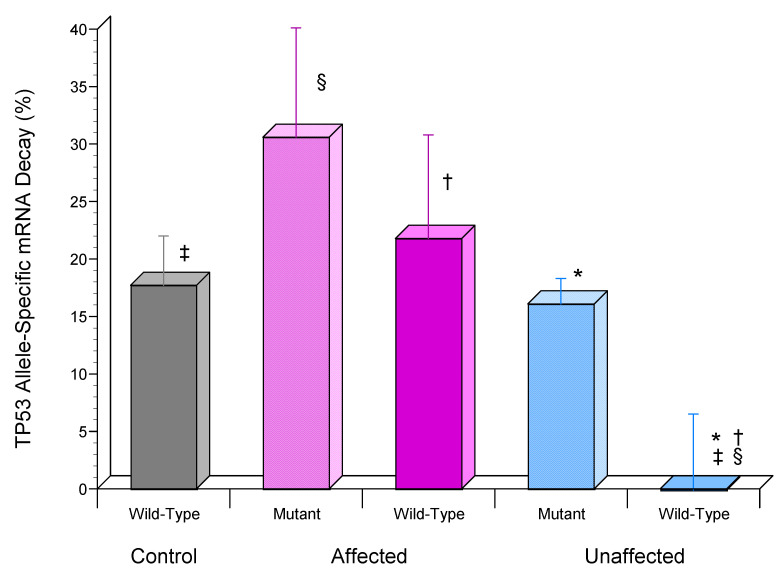
Wild-type *TP53* allele-specific mRNA stabilization in dFb from unaffected compared to affected LFS or control subjects following UV exposure. RT-PCR-RFLP assay customized to detect the heterozygous Ser241Tyr C > A *TP53* mutation in this LFS family was used to calculate proportions of wild-type and mutant *TP53* mRNA remaining 6 h after transcriptional inhibition with actinomycin D following UV irradiation, as described (*n* = 4: * *p* < 0.05, unaffected wild-type vs. unaffected mutant; ^†^ *p* < 0.05, unaffected wild-type vs. affected wild-type; ^§^ *p* < 0.05, unaffected wild-type vs. affected mutant; ^‡^ *p* < 0.05, unaffected wild-type vs. wild-type control). Expression of *TP53* allele mRNA was normalized relative to *B2M* mRNA as a stable reference standard.

Additionally, this finding suggested that the previously reported preferential expression of the wild-type *TP53* allele by the unaffected dFb may be regulated by its allele-specific mRNA stabilization. To investigate this possibility, the custom RT-PCR-RFLP assay previously designed to detect the *TP53* mutation in this LFS family [18] was modified to measure allele-specific mRNA decay by including amplification of *B2M* as a stable reference standard [23,25]. This analysis found that, while 21.8 ± 9.0% and 17.7 ± 4.3% of wild-type *TP53* mRNA had decayed after 6 h in affected and control dFb, respectively, essentially none (−0.1 ± 6.6%) had decayed in the unaffected dFb (Figure 6), as was also the case for total *TP53* mRNA (Figure 5). Although less of the mutant *TP53* mRNA also appeared to be decayed in the unaffected (16.1 ± 2.2%) vs. the affected (30.6 ± 9.5%) dFb, this difference (Figure 6) was not significant (*p* > 0.05). Additionally, the difference between wild-type (21.8 ± 9.0%) and mutant (30.6 ± 9.5%) *TP53* mRNA decay in the affected dFb (Figure 6) was not significant either (*p* > 0.05).

## 4. Discussion

This study was designed to further characterize the potential dysregulation of cell cycle arrest [17] and *TP53* allele-specific expression [20] previously reported for fibroblasts from an LFS unaffected carrier compared to his affected offspring in response to UV stress [18]. Our previous study suggested that the reduced UV induction of *CDKN1A*/p21 mRNA and protein expression by the affected dFb [18] could dysregulate p21-dependent cell cycle arrest response to UV stress [47], as has also been reported in affected dFb with other *TP53* mutations [17].

Therefore, the first portion of the present study was designed to examine the signal transduction pathway regulating the cell cycle response to UV exposure. Phosphorylation of the cell cycle checkpoint kinases 1 (Chk1) and 2 (Chk2) [30], substrates of the key DNA damage protein kinase sensors, ATR and ATM, respectively [29], was selected for this analysis. This comparison found significantly increased phospho-Chk1[S345] in protein extracts from the affected dFb compared with both the unaffected and control dFb following UV exposure (Figure 1), whereas no significant UV-induced phospho-Chk2[T68] was found in any of the dFb tested. Unexpectedly, this UV-induced increase in phospho-Chk1[S345] was found to potentially be reinforced by the previously observed UV-induced phospho-p53[S15] upregulation in the affected dFb [18], since it has been shown that phospho-p53[S15] may play a role in sustaining activated phospho-Chk1[S345] [33].

In short, this increased phospho-Chk1[S345] would suggest a stronger cell cycle arrest response to UV exposure [48] in the affected dFb (Figure 1). Such a response would appear to potentially be at odds with the reduced UV induction of *CDKN1A*/p21 mRNA and protein expression by the affected dFb found in previous studies [17,18], which was suggested to be indicative of an aberrant p21-dependent cell cycle arrest response [47]. However, p21 is a multifunctional stress response factor regulating a variety of other key cell cycle processes, most notably senescence and apoptosis [49]. Hence, it is possible that, while UV activation of the ATR-Chk1 pathway may induce a more potent cell cycle arrest response to facilitate DNA repair in the affected LFS dFb [33], the accompanying reduced induction of *CDKN1A*/p21 in those affected dFb [17,18] may counteract this repair effort by impairing senescence or apoptosis of those cells with unrepaired DNA damage [49]. Additional experiments are underway to assess the mechanism of this potential cell cycle dysregulation in greater detail. In short, the upregulation of phospho-p53[S15] [18] and phospho-Chk1[S345] (Figure 1) combined with the reduced expression of p21 [17,18] in UV-induced affected dFb vs. unaffected and control dFb represent a striking irregularity that may play a key role in the onset of cancer.

The next portion of this study was designed to characterize properties of the p53 protein expressed by unaffected compared to affected dFb in response to UV exposure. First, immunoblot analysis with a commercial antibody developed to detect mutant p53 was performed to determine if the decreased mutant *TP53* allele-specific mRNA expression reported previously in the unaffected dFb [18] translated into decreased expression of mutant p53 protein, as well. This was, in fact, found to be the case (Figure 2). Next, the p53 promoter binding activity of UV-induced nuclear extracts from the affected dFb vs. both the unaffected and control dFb was compared using an activity assay to measure p53 affinity for a consensus promoter binding site [37]. The resultant finding that protein extracts from the affected dFb had significantly higher p53 binding with the target consensus promoter element than that of the unaffected or the control dFb (Figure 3) may seem contrary to expectations, based on the atypical binding domain of the higher mutant p53 expressed by the affected dFb (Figure 2). However, the promoter binding affinity of mutant [35,36] and phosphorylated [19,34] p53 is not so predictable and can depend upon the specific target binding site as well [50], especially since there is considerable ambiguity in its consensus sequence [37]. In fact, this flexibility appears integral to the role of p53 in activating the complementary genetic programs for cell cycle arrest and/or apoptosis in response to genotoxic stress, such as UV exposure [1,35,51]. Taken together, the increased mutant p53 expression (Figure 2) and/or S15-phosphorylation [18] in protein extracts from the affected dFb are likely to play a role in regulating its promoter binding activity (Figure 3). Indeed, the reduced UV induction of *CDKN1A*/p21 expression [17,18] by the affected dFb observed in our previous study may be regulated by the altered binding affinity and/or specificity of mutant p53 [47,49] in these dFb (Figure 2 and Figure 3).

The final portion of this study was focused on investigating the mechanism regulating allele-specific expression of wild-type *TP53* previously found in the heterozygous dFb from the unaffected parent [18]. Our initial hypothesis [18] that it may be regulated by genomic imprinting [38] via differential *TP53* promoter methylation [39] was found not to be the case. Methylation of the *TP53* promoter regions from −1654 to +54 was undetectable in gDNA from any of these subjects, despite the presence of a canonical CpG island [39]. Since a prominent role for mRNA stability in regulating *TP53* expression has also been described [42,43,44,45,46], *TP53* mRNA half-life was then compared in UV-induced unaffected dFb vs. affected and control dFb to determine its possible contribution to the higher UV-induced total *TP53* mRNA expression found in the unaffected dFb (Figure 4). The strikingly high stability of UV-induced total *TP53* mRNA expression by the unaffected dFb that was observed throughout the duration of the study (Figure 5) very likely contributed considerably to the upregulation of total unaffected dFb *TP53* mRNA expression (Figure 4). Moreover, the subsequent analysis of *TP53* allele-specific mRNA stability revealed that this high stability of total *TP53* mRNA in the unaffected dFb (Figure 5) was reflected in the novel, specific stability of the wild-type *TP53* mRNA, which was also uniquely stable in these cells throughout the study duration (Figure 6).

These results support the hypothesis that allele-specific stabilization of wild-type *TP53* mRNA (Figure 6) likely plays a role in regulating both the higher total (Figure 4) and preferential wild-type [18] *TP53* mRNA expression in the unaffected dFb. Studies to further investigate the detailed mechanism regulating this remarkable allele-specific *TP53* mRNA stability are underway. Stabilization of the wild-type *TP53* mRNA would appear to be central for the maintenance of wild-type p53 functional tumor suppression in the heterozygous unaffected LFS carrier.

## Data Availability

Data presented available on request from corresponding author. Due to patient privacy, data not publicly available.
